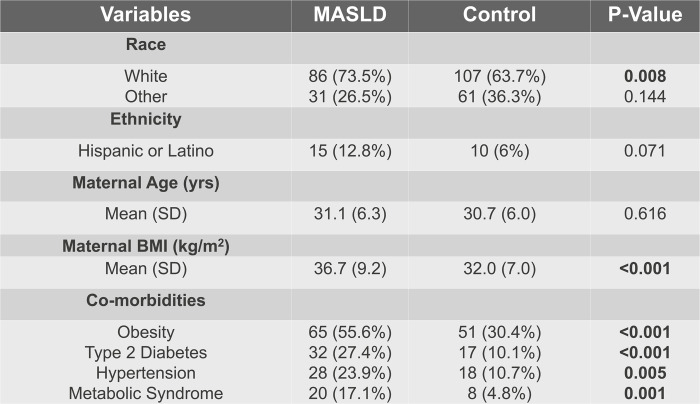# Pregnancy does not affect liver chemistries in metabolic dysfunction–associated steatotic liver disease

**DOI:** 10.1097/HC9.0000000000000587

**Published:** 2024-11-25

**Authors:** Zoe Finer, Christine Lopez, Suzanne Sharpton, Yue Gao, Christopher Lindsell, Rolanda Lister, Jennifer Thompson, Manhal Izzy

**Affiliations:** 1 Vanderbilt University School of Medicine, Nashville, Tennessee, USA; 2 Division of Gastroenterology, Department of Medicine, Hepatology, and Nutrition, Vanderbilt University Medical Center, Nashville, Tennessee, USA; 3 Vanderbilt Institute for Clinical and Translational Research Methods Program, Nashville, Tennessee, USA; 4 Division of Maternal-Fetal Medicine, Department of Obstetrics and Gynecology, Vanderbilt University Medical Center, Nashville, Tennessee, USA

**Keywords:** metabolic dysfunction–associated steatotic liver disease, metabolic syndrome, pregancy, insulin-resistance, transaminases

## INTRODUCTION

Metabolic dysfunction–associated steatotic liver disease (MASLD) is an increasingly common cause of chronic liver disease with an estimated prevalence of 38% in the United States.^1^ Young adults (18–40 y) have seen the most significant rise in MASLD, with an increase of 2.5 times between 1988 and 2010^2^, reflecting the worsening of the obesity epidemic.^3^ MASLD and metabolic syndrome often co-exist and share overlapping key pathophysiological mechanisms, including inflammation, insulin resistance, and genetic predisposition.^4^

Reproductive health implications of MASLD should be considered, especially with the increasing prevalence of MASLD among reproductive-aged women of up to 20%.^2^^,^^5^ While the effects of pregnancy on MASLD are not well defined, MASLD and metabolic syndrome have been suggested to be associated with adverse maternal outcomes.^5^^,^^6^ Importantly, pregnancy shares similar physiology to some key drivers of MASLD development, including insulin resistance and oxidative stress.^6^ Further, the risk of metabolic complications is intensified during pregnancy due to increased visceral adiposity, including intrahepatic lipid deposition.^6^ We hypothesized that pregnant patients with MASLD are at higher risk for the development of worsening liver inflammation, as reflected by changes in transaminases, compared to pregnant patients without MASLD.

## METHODS

This is a retrospective study of all adult (>18 y) pregnant patients at a tertiary North American center between 2000 and 2021. Diagnostic codes and steatosis-indicative keywords from radiology reports identified patients with MASLD before pregnancy to compare to age-matched controls without liver disease (Supplemental Appendix 1, http://links.lww.com/HC9/B90). Of note, after data collection completion for this study, there was a nomenclature shift from NAFLD to MASLD. MASLD reflects the associated cardio-metabolic abnormalities and removes the exclusion criteria of preexisting liver disease. However, as outlined above, our study used diagnostic codes and radiology reports to identify cases, given the NAFLD definition during the study period. Patients without documented AST, ALT, and platelet values within 2 years before conception were excluded. For pre-pregnancy transaminase values, the value closest to the date of pregnancy was used. For during-pregnancy transaminase values, the highest value during pregnancy was used. Fibrosis-4 Index was assessed as a noninvasive biomarker of fibrosis severity in patients with MASLD.^7^ Multivariate linear regression was used for the evaluation of transaminases during pregnancy, and logistic regression was used for categorical outcomes.

## RESULTS

Two hundred eighty-five patients were included, with 117 MASLD cases and 168 controls in a 1:1.5 age-matched ratio. Most patients in both groups were White (Supplemental Table S1, http://links.lww.com/HC9/B91). Hispanic or Latino patients had increased rates of MASLD (Supplemental Table S1, http://links.lww.com/HC9/B91). Patients with MASLD had significantly increased pre-pregnancy comorbid conditions (Supplemental Table S1, http://links.lww.com/HC9/B91). Obesity based on pre-pregnancy BMI was more severe in the MASLD group (MASLD 36.7 [9.2], Control 32.0 [7.0], *p* < 0.01) (Supplemental Table S1, http://links.lww.com/HC9/B91). Patients with MASLD had significantly higher pre-pregnancy transaminases compared to controls. However, transaminases were comparable between the 2 groups during pregnancy, as were the mean differences during pregnancy compared to pre-pregnancy levels (Table 1). In multivariate analysis, MASLD (*p* = 0.39), pre-pregnancy AST (*p* = 0.65), and the interaction between MASLD and pre-pregnancy AST (*p* = 0.60) were not associated with AST levels during pregnancy.

**TABLE 1:** Descriptive Statistics for Pre-Pregnancy and During Pregnancy AST and ALT

	MASLD vs. control			MASLD subgroups (based on FIB-4)		
	MASLD (mean, SD) (n = 117)	Control (mean, SD) (n = 168)	*p*	At risk of advanced fibrosis (mean, SD) (n = 25)	Not at risk of advanced fibrosis (mean, SD) (n = 92)	*p*
Pre-pregnancy AST	30.7 (38.7)	22.1 (8.65)	0.02	48.0 (68.7)	26.0 (23.7)	0.127
Pre-pregnancy ALT	34.9 (57.1)	19.3 (14.6)	0.005	45.3 (43.1)	32.1 (60.2)	0.221
During-pregnancy AST	29.8 (52.9)	24.0 (9.50)	0.319	25.1 (7.07)	31.4 (61.2)	0.42
During-pregnancy ALT	26.2 (36.8)	18.1 (9.32)	0.069	22.3 (14.5)	27.5 (41.9)	0.413
Difference between during-pregnancy and pre-pregnancy AST	1.87 (59.4)	3.03 (10.3)	0.86	−13.0 (42.6)	7.00 (63.6)	0.102
Difference between during-pregnancy and pre-pregnancy ALT	−6.66 (61.6)	0.509 (11.4)	0.322	−18.3 (33.5)	−2.58 (68.6)	0.186

Abbreviations: FIB-4, Fibrosis-4 Index; MASLD, metabolic dysfunction–associated steatotic liver disease.

Similarly, MASLD (*p* = 0.31), pre-pregnancy ALT (*p* = 0.70), and the interaction between MASLD and pre-pregnancy ALT (*p*=0.75) were not associated with ALT levels during pregnancy. In a subgroup analysis of patients with MASLD, at risk for advanced fibrosis (defined by having intermediate or high Fibrosis-4 Index or having co-occurring metabolic syndrome) was not associated with AST or ALT trend during pregnancy (AST: *p* = 0.71, ALT: *p* = 0.50). Finally, on univariate analysis, there were no significant differences in pregnancy-related outcomes, including gestational hypertension (*p* = 0.14), gestational diabetes (*p* = 1.0), and pre-eclampsia (*p* = 0.63) between the study groups.

## DISCUSSION

In our cohort, neither MASLD nor its severity, determined by Fibrosis-4 Index, was associated with the trends of transaminases during pregnancy. Based on our results, changes in transaminases during pregnancy should not be assumed to be MASLD-related, despite the pregnancy-associated metabolic changes. Our findings emphasize the importance of a comprehensive evaluation of liver biochemical abnormalities during pregnancy as they may reflect evolving pregnancy-related liver disease in the setting of concurrent MASLD. To our knowledge, this is the first study analyzing pregnancy’s effect on transaminases in patients with pre-pregnancy MASLD.

Although this cohort was extracted out of 4.7 million screened medical records spanning 2 decades, the sample size is limited as we excluded patients without transaminases documented within 2 years of conception. However, of note, elevated liver chemistries may not accurately reflect hepatic inflammation as patients with normal liver chemistries can still have a degree of steatohepatitis.^8^ Both groups had patients with metabolic risk factors for MASLD, reflective of the general population. We did not exclude control patients with MASLD risk factors to preserve study generalizability. While the control group may have included patients with undiagnosed MASLD, multiple methods to identify patients with MASLD, including problem list documentation, radiology reports, and diagnostic codes, were used. Pre-pregnancy laboratory testing demonstrated higher baseline transaminases in the MASLD group, and 30% (50/168) of control patients had abdominal imaging, which did not show hepatic steatosis. Both observations validate our methodology for classifying cases versus controls. Patients with other etiologies of liver disease, including but not limited to pregnancy-related liver disease, based on ICD codes and problem lists, were excluded.

As there were no clinical practice guidelines specific to MASLD in pregnancy during the study period, the MASLD group was managed similar to control patients unless they had other comorbidities, such as obesity, that necessitated specialized care. For example, obese patients require earlier screening for metabolic disorders and increased fetal monitoring based on clinical practice guidelines.^9^

## CONCLUSIONS

Understanding pregnancy’s effect on MASLD is important for preconception and antenatal counseling. Our findings suggest that pregnancy physiology does not significantly exacerbate liver inflammation as reflected by lack of significant changes in liver chemistries during pregnancy in patients with pre-existing MASLD. Elevation in transaminases among pregnant patients with MASLD should be investigated rather than being attributed to MASLD. This work provides the foundation for future multicenter prospective studies to examine the effects of pregnancy physiology on MASLD.

## Supplementary Material

**Figure:**