# Supplementary material for: Pregnancy does not affect liver chemistries in metabolic dysfunction–associated steatotic liver disease
Source: Hepatol Commun. 2024 Nov 25;8(12):e0587. doi: 10.1097/HC9.0000000000000587 (PMC11596671; doi:10.1097/HC9.0000000000000587)
Supplement: Supplementary file 1 [file hc9-8-e0587-s001.docx]

Supplementary Appendix 1:

Diagnostic codes and steatosis-indicative keywords from radiology reports identified patients with MASLD prior to pregnancy to compare to age-matched controls without liver disease. The diagnostic codes included ICD9 571.8 as well as ICD10 K75.8 and K76.0. The key words from patients’ problem lists to identify MAFLD were NAFLD, NASH, hepatic steatosis, and fatty liver. The key words in radiology reports to identify MAFLD were Hepatic steatosis, fatty infiltration, fatty liver, increased hepatic echogenicity, and increased liver attenuation.
